# Biphasic synaptic plasticity of the medial thalamic-cingulate pathway contributes to pain hypersensitivity in vivo

**DOI:** 10.1177/17448069261466896

**Published:** 2026-07-15

**Authors:** Shen Lin, Shun Hao, Yi Wu, Weiqi Liu, Jihua Wang, Le Li, Wanfen Liu, Min Zhuo, Wucheng Tao

**Affiliations:** 1 Fujian Key Laboratory of Cognitive Function and Diseases, 74551 Fujian Medical University, Fuzhou, Fujian, China; 2 Fujian Provincial Institutes of Brain Disorders and Brain Sciences, First Affiliated Hospital, 74551 Fujian Medical University, Fuzhou, Fujian, China; 3 Key Laboratory for Space Bioscience and Biotechnology, School of Life Science and Technology, 26487 Northwestern Polytechnical University, Xi’an, Shanxi, China; 4 Department of Physiology, Faculty of Medicine, 7938 University of Toronto, Toronto, ON, Canada

**Keywords:** synaptic plasticity, pain, chronic pain, nociception, thalamus, anterior cingulate cortex, *in vivo*, theta burst stimulation, low-frequency stimulation, long-term potentiation, long-term depression, allodynia

## Abstract

Central synaptic plasticity is a key mechanism for long-term memory and chronic pain. The anterior cingulate cortex (ACC), a critical cortex area for pain perception, receives sensory inputs from the thalamus. Although many studies have demonstrated that synaptic transmission in the ACC is potentiated in different chronic pain models, there are few studies investigating synaptic changes of the thalamus-ACC pathway in vivo. Here we selectively stimulated the thalamus-ACC pathway by using optogenetic approach in adult mice both in vitro and in vivo. Theta burst stimulation (TBS) induced robust and long-lasting long-term potentiation (LTP) in the medial thalamus-ACC pathway in the ACC of adult mice in vitro. The potentiation lasted for at least 2 to 3 hrs after the induction, and some of silent responses were recruited. In freely moving adult mice, the same stimulation of the medial thalamus-ACC pathway induced rapid and long-lasting potentiation of synaptic responses in the ACC of adult mice in vivo. This potentiation lasted for days and weeks, at least six weeks after the induction in vivo. In parallel, behavioral sensory responses to noxious mechanical and thermal stimuli were sensitized in the same animals while anxiety-like behaviors were not significantly affected. Furthermore, low-frequency stimulation induced long-term depression (LTD) in the ACC, and reversed mechanical and thermal sensitization in animals with LTP. Our results provide strong evidence that biphasic synaptic plasticity (LTP and LTD) of the thalamus-ACC pathway contributes to behavioral sensitization after peripheral injury, and understanding the mechanisms for such LTP may help future treatment of chronic pain in patients and pets.

## Introduction

Pain is transmitted from the periphery to the brain through several key central relays including the spinal cord dorsal horn and thalamus. After periphery injuries such as inflammation or nerve injury, it is well known that glutamatergic synapses in the spinal cord including spinothalamic tract (STT) cells are sensitized or undergo long-term potentiation (LTP).^1,2^ The anterior cingulate cortex (ACC) plays critical roles in pain perception and related emotional responses.^3–9^ Human imaging and in vivo electrophysiological recordings of animal ACC neurons show that neurons in the ACC are activated by noxious sensory stimuli,^10,11^ and inhibiting central plasticity in the ACC produces analgesic effects in different animal models of chronic pain.^8,12^

Synaptic plasticity, LTP, is critical for memory, fear and chronic pain.^8,12–15^ Cumulative evidence suggests that ACC synapses are highly plastic. Two major forms of LTPs have been found in the ACC: pre-LTP and post-LTP in vitro. Post-LTP in the ACC is typically induced by theta burst stimulation (TBS).^12^ The activation of postsynaptic NMDA receptors is critical for the induction of post-LTP, while postsynaptic modification of AMPA receptor contribute to the expression of post-LTP. Behavioral and genetic studies suggest that post-LTP preferentially contribute to behavioral sensitization after peripheral injury, pre-LTP contributes injury-related anxiety.^12,16^ It is generally believed that ACC neurons receive peripheral sensory information through thalamus-ACC projections as well as indirect projections from somatosensory cortices.^17–19^ In addition, recent studies reveal that ACC neurons also receive projections from hippocampus, amygdala, and other subcortical areas.^17,18^ Due to the limitation of focal electrical stimulation, previous studies did not distinguish the selective input source in vitro brain slice and in vivo electrophysiological experiments.^20–22^ Furthermore, few studies of ACC LTP is recorded from freely moving animals.

Thalamus is a key relay nucleus for transferring spinal nociceptive information to various cortical regions (by STT cells).^23,24^ The projection from the thalamus to the ACC have been well documented in different species, and it is believed to be major nociceptive inputs to the ACC, in addition to cortical projections from somatosensory cortice.^7,12,25^ Electrophysiological experiments using *in vivo* thalamic stimulation in adult rats have confirmed the existence of thalamic-anterior cingulate projections. Electrical stimulation of mediodorsal (MD), midline, and intralaminar thalamic nuclei induced short-term plastic changes in layers II/III of the ACC that transmit nociceptive information to the ACC in early stage of chronic pain.^25,26^ However, there is no report of LTP in these studies. Recently in adult mice we have investigated the projection to ACC and showed direct monosynaptic glutamatergic connections from the thalamus to ACC pyramidal cells in vitro by using optogenetic stimulation.^17,18^

In addition to sensory pain perception, recent studies have also shown that ACC neurons/synapses could contribute to fear, social pain, sleep disorders, depression and memory. Different ACC-related functions are likely mediated through different anatomic projections with other cortical and subcortical areas.^27–30^ Thus, it becomes necessary to distinguish the presynaptic inputs in the study of slice physiology. Due to the limit of technology in vitro, most of studies of ACC LTP or LTD use focal electrode stimulation and it is hard to know the types of presynaptic fibers are activated in these experiments.^16,31^ Secondary, the duration of ACC LTP is mostly done in in vitro brain slices, and lasted for a few hours.^12,32^ There is lack of evidence that such LTP may last for several days or weeks when behavioral hyperalgesia was observed.^12,31,33^ While indirect evidences were reported in studies using in vitro brain slices prepared from injured animals,^20,32^ direct evidence for in vivo LTP is still lacking. Finally, while many reports suggest that excitatory inputs from the thalamus to the ACC are likely to undergo potentiation in condition of chronic pain,^12^ there is no direct evidence that the thalamus-ACC pathway is plastic under in vitro and in vivo conditions.

In this study, we hypothesize that the thalamus-ACC pathway is plastic in adult animals, and its LTP could contribute to chronic pain. Furthermore, we wanted to examine if TBS induced LTP, a postsynaptic form of LTP, may be specific for behavioral sensory and/or anxiety-like responses. By combining the multi-channel field potential recording and optogenetic technique, we report a long-lasting LTP in the thalamus-ACC projection in vitro. By performing in vivo electrophysiological recording, we show that TBS induced long-lasting LTP of the thalamus-ACC pathway in vivo, from several hours to days. Behaviorally, TBS induced LTP of the thalamus-ACC caused behavioral sensitization without significant changes in anxiety-like responses.

## Results

### TBS in the mTH-ACC pathway induced LTP in ACC in vitro

**Figure 1.**
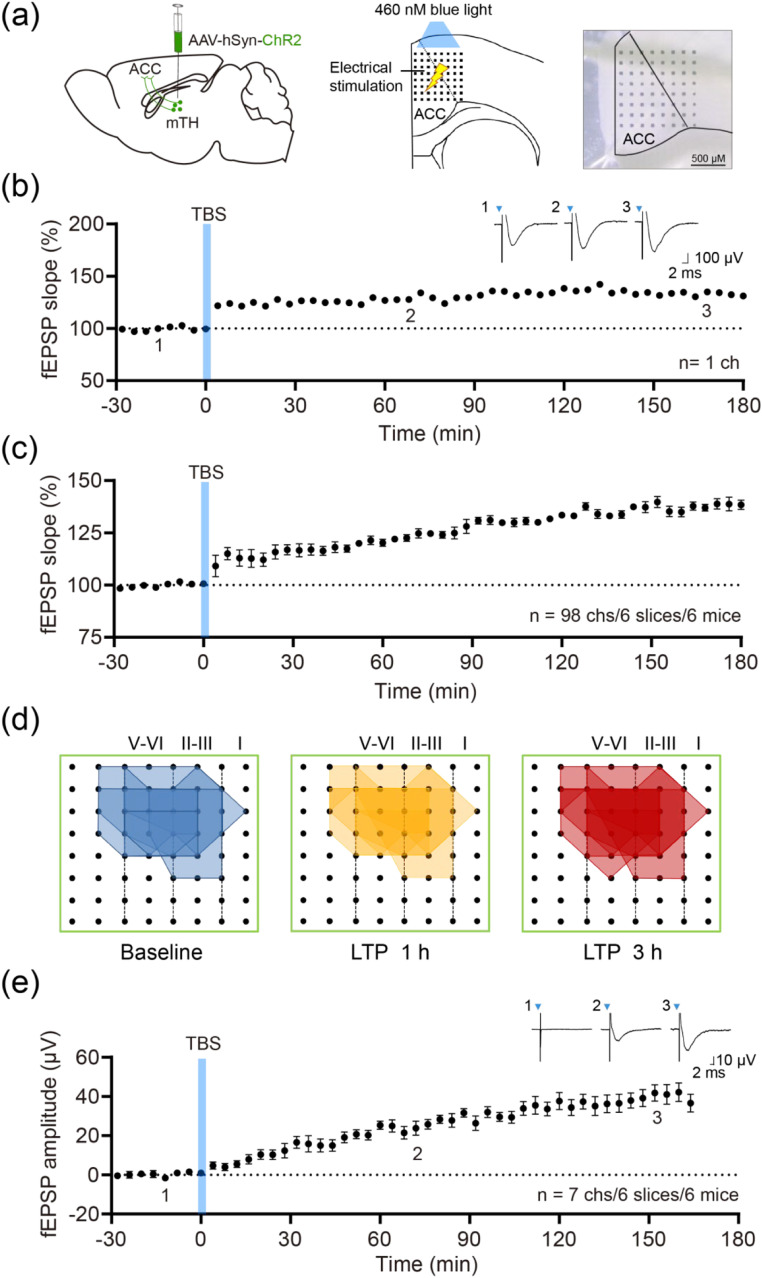
*TBS in the mTH-ACC pathway induced local LTP in the ACC in vitro*. (a), Schematic (left) of optogenetic viral (AAV-hSyn-ChR2(H134R)-EGFP) injection site in the mouse brain. Schematic and microscopic image (right) of the relative position between the MED-64 probe and the ACC in the brain slice. The fEPSPs were evoked by electrical stimulation from one site of the MED-64 probe. (b), Top, representative fEPSP traces recorded at the different time points indicated by the numbers in the curve below. Bottom, the example curve of fEPSP slope from one channel showed the formation of LTP lasting for at least 3 hours induced by theta-burst blue light stimulation. The blue bar indicated the timepoint of lighting. (c), Pooled data showed the changes of the fEPSP slope with the induction of persistent LTP by light stimulation that lasts for 3 hours (n = 98 chs/6 slices/6 mice). (d), The polygonal diagrams showed the distribution of the activated channels in the ACC in the baseline (blue), 1-hour (orange), and 3-hour (red) phase (n = 6 slices/6 mice). (e), The temporal changes of the fEPSP amplitude from recruited channels in the ACC after theta-burst light stimulation (n = 7 chs/6 slices/6 mice).

Previous studies using *in vitro* brain slices from adult rats and mice have consistently shown that excitatory synapses in the ACC are highly plastic. TBS is able to induce long-lasting LTP that persists for at least 6 h.^8,12^ In most of these studies, focal stimulation was delivered within the ACC, and it remains unclear whether activation of the mTH-ACC pathways could also induce LTP in this region. First, we wanted to investigate whether LTP can be induced by blue light stimulation following optogenetic activation of the ChR2-expressing mTH-ACC projection fibers in ACC slices. To induce baseline responses, we stimulated a synaptic input at one site within the ACC and simultaneously recorded the multi-channel field excitatory postsynaptic potentials (fEPSPs; Figure 1(a)) as reported previously.^32–34^ Consistent with previous studies, reliable responses were elicited from local stimulation sites. After obtaining a stable baseline for 30 min, we delivered TBS. TBS induced long-lasting potentiation of fEPSPs (see example in Figure 1(b)). The potentiation persisted for at least 3 h following the induction (mean 140.5 ± 5.8% of baseline in last 20 min, n = 6 slices/6 mice, *p*= 0.0001, paired *t*-test, Figure 1(c)).

Our previous studies have consistently reported the recruitment of ‘silent’ responses after ACC LTP.^33–35^ As we predict, TBS in the mTH-ACC pathway also recruits ‘silent’ responses. Some channels (n = 7 channels from total 6 slices/6 mice, Figure 1(d)) that were inactive during baseline recordings showed evoked fEPSPs after the TBS. Following induction, the average amplitude of fEPSPs in these recruited channels gradually increased (finally reaching 40.2 ± 4.9 μV) and remained stable for over 2 h (Figure 1(e)). Our field recording cannot determine if such recruitment is due to the activation of silent synapses; the study by Vardalaki et al. have demonstrated that silent synapses do exist in adult somatosensory cortical regions.^36^ These findings suggest that activation of the mTH-ACC pathways may similarly engage silent synapses in the adult brain.

**Figure 2.**
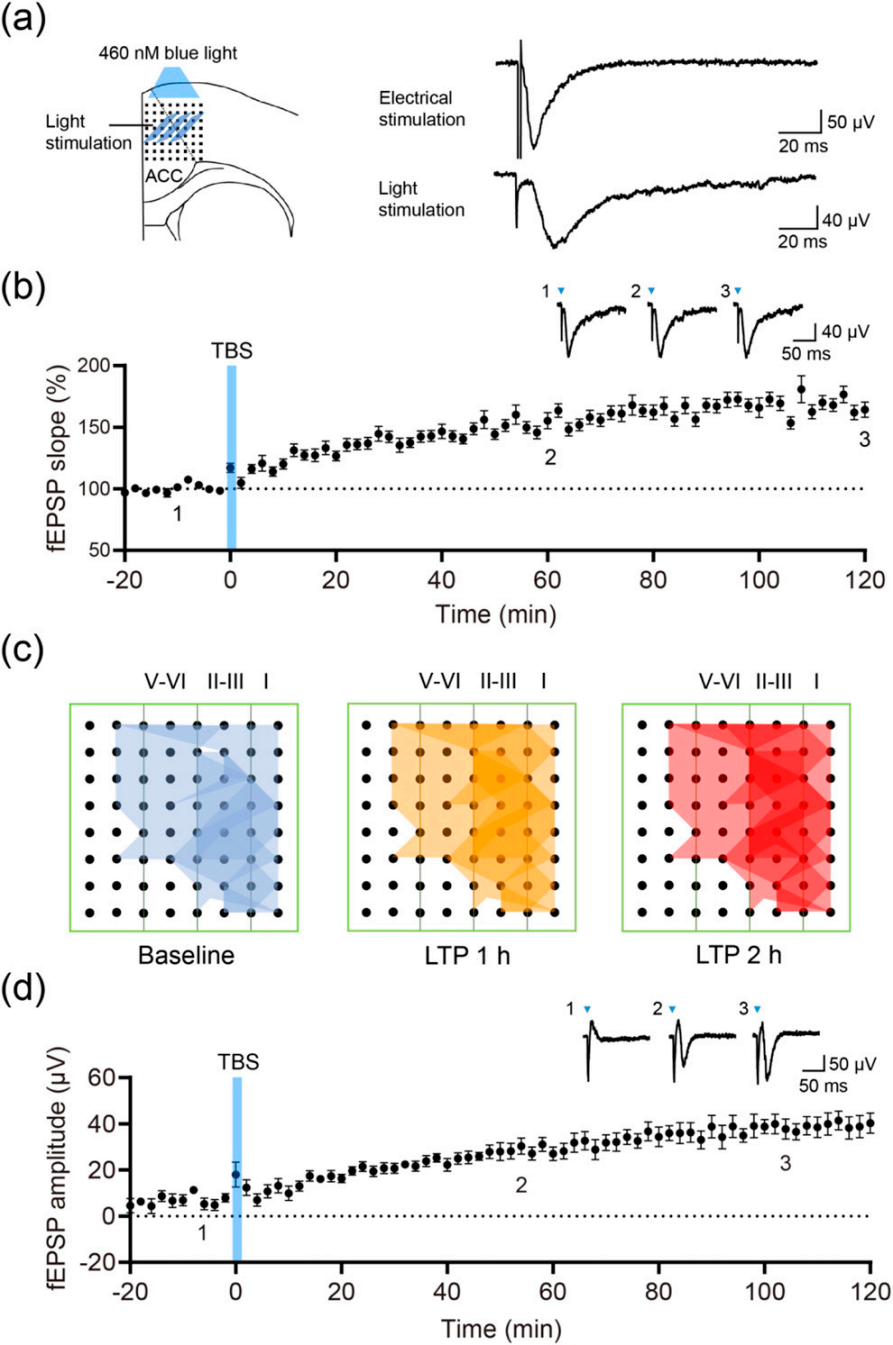
*TBS induced LTP in the mTH-ACC pathway by optogenetic manipulation in vitro*. (a), The schematic (left) showing the fEPSPs evoked by blue light stimulation (10-30 mW, 0.2-0.5 ms) in the mTH-ACC pathway. The example traces of single fEPSP evoked by electrical (upper) or light (bottom) stimulation. The peak latency of the fEPSP evoked by electrical stimulation was 7.4 ms. The peak latency of the fEPSP evoked by light stimulation was 17.6 ms. (b), The summarized fEPSP slopes from the channels showed the induction of LTP lasting for 2 hours after TBS in the mTH-ACC pathway (n = 78 chs/5 slices/4 mice). (c), The polygonal diagrams showed the distribution of the activated channels in the mTH-ACC pathway in the baseline (blue), 1-hour (orange), and 2-hour (red) phase (n = 4 slices/3 mice). (d), The temporal changes of the fEPSP amplitude from recruited channels in the mTH-ACC pathway after TBS (n = 12 chs/4 slices/3 mice).

To further verify that LTP was induced in the mTH-ACC pathway, single-pulse electrical stimulation was replaced by light stimulation to evoke fEPSPs (Figure 2(a) left). Single-pulse light stimulation reliably triggered fEPSPs with normal amplitudes similar to that by focal electrical stimulation (Figure 2(a), right). Similarly, TBS in the mTH-ACC pathway induced LTP in the ACC. The potentiation lasted for at least 2 h (mean 168 ± 5.3% of baseline in last 20 min, n = 5 slices/4 mice, *p*= 0.0001, paired t-test, Figure 2(b)). Furthermore, we also found the recruitment of silent responses after TBS, the average recruited responses reached mean 39.1 ± 4.2 μV (n = 12 channels from total 4 slices/3 mice, Figure 2(c) and (d)). These results indicate that TBS in the mTH-ACC pathway induces LTP, and recruits ‘silent’ responses in the ACC in vitro.

### Recording ACC responses to stimulation of the mTH-ACC pathway in freely moving mice

**Figure 3.**
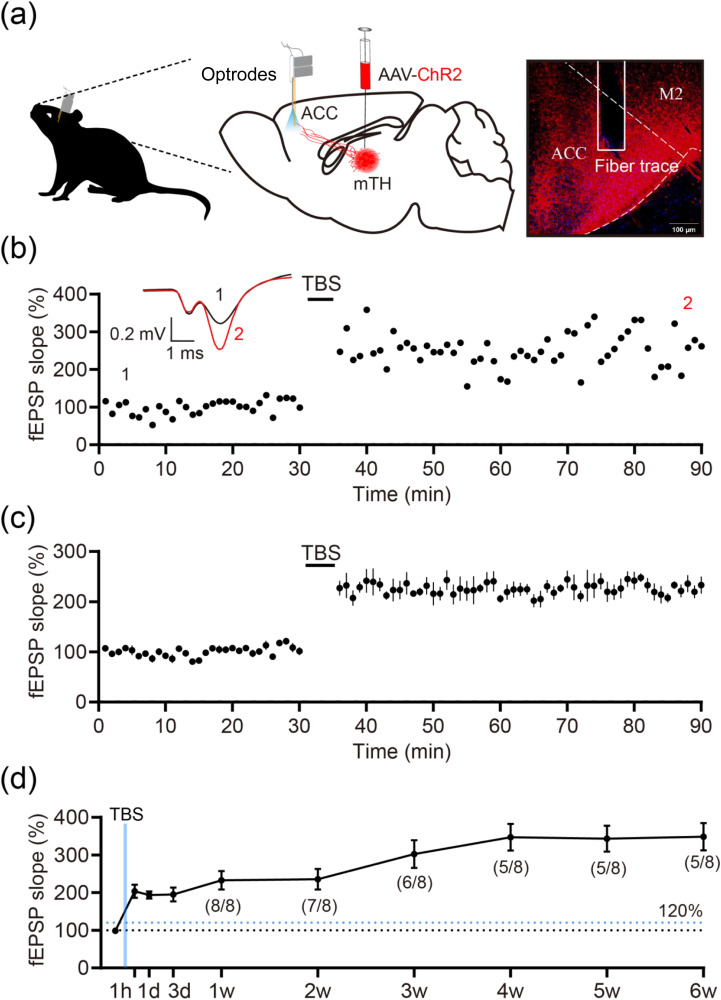
*TBS produced long-lasting LTP in the mTH-ACC pathway in vivo*. (a), Schematic (left) of optogenetic viral injection. Example placement of optrode (right). Red, terminals expressing mCherry from mTH; blue, DAPI. Dashed white lines are boundaries of subregions. (b), Top, representative fEPSP traces recorded at the different time points indicated by the numbers in the curve below. Bottom, the example curve of fEPSP slope induced by theta-burst blue light stimulation. (c), Average of 6 experiments (n = 6 mice, down) of field EPSP slope (normalized to baseline period) before and after indicated stimulation. Scale bars: 0.2 mV, 1 ms. Error bars show s. e.m. (d), Normalized field EPSP slope (n = 8 mice, normalized to baseline period) after TBS up to 6 weeks. The blue bar indicated the timepoint of lighting.

It is generally believed that neurons in the ACC neurons receive sensory inputs though the thalamic projections including the mTH.^7,8,25^ Our recent study in adult mice confirmed that the mTH-ACC pathway is the major thalamic projection to the ACC, and this fast excitatory connection is mediated by glutamatergic AMPA receptors.^17^ In order to characterize the synaptic transmission and plasticity of the mTH-ACC pathway in freely moving mice, we first injected an adeno-associated virus expressing channelrhodopsin-2 (AAV-ChR2-mCherry) into the right mTH (see Figure 3(a)). We confirmed that there was significant amount of expression of ChR2 in the mTH neurons and their projecting axons in the ACC (Figure. S1). Subsequently, we implanted optrodes into the right ACC to measure the synaptic responses to the activation of the mTH-ACC projection (n= 3 mice; Figure S1). The excitatory field responses were induced by the blue light, and such responses were stable for a few hours to days (n =3 mice; 96.0±6.2% for day 2 and 101.0±6.6% for day3 of the baseline in day 1).

### LTP induced by TBS in vivo

Previous in vitro brain slice experiments have consistently demonstrated that TBS induced robust LTP in ACC slices of adult mice.^12,31,32^ There is no report of ACC LTP in freely moving animals so far, to our knowledge. Next, we wanted to test the ACC LTP after TBS in vivo in freely moving mice. After obtaining stable baseline responses, we then delivered TBS to the mTH-ACC pathway using blue light activation. We found that the field responses were significantly potentiated immediately after the application of TBS (n=6 mice, P<0.001 as compared with a mean 208.0±7.9% of the baseline, Figure 3(b) and (c)), and the potentiation lasted for at least 1 hour (n= 6 mice).

To determine the duration of TBS induced L-LTP, we performed long-term recordings from weeks to months without additional interventions in total of 8 mice. All of 8 mice showed the significant LTP (magnitude change >120%), the magnitude of fEPSPs became gradually stronger 1-2 weeks after TBS (mean 187%; n=8 mice). At six weeks after the induction, we found significant potentiation in 5/8 mice while the other three mice returned to normal (Figure 3(d)).

### Behavioral sensitization after the induction of the mTH-ACC LTP in vivo

**Figure 4.**
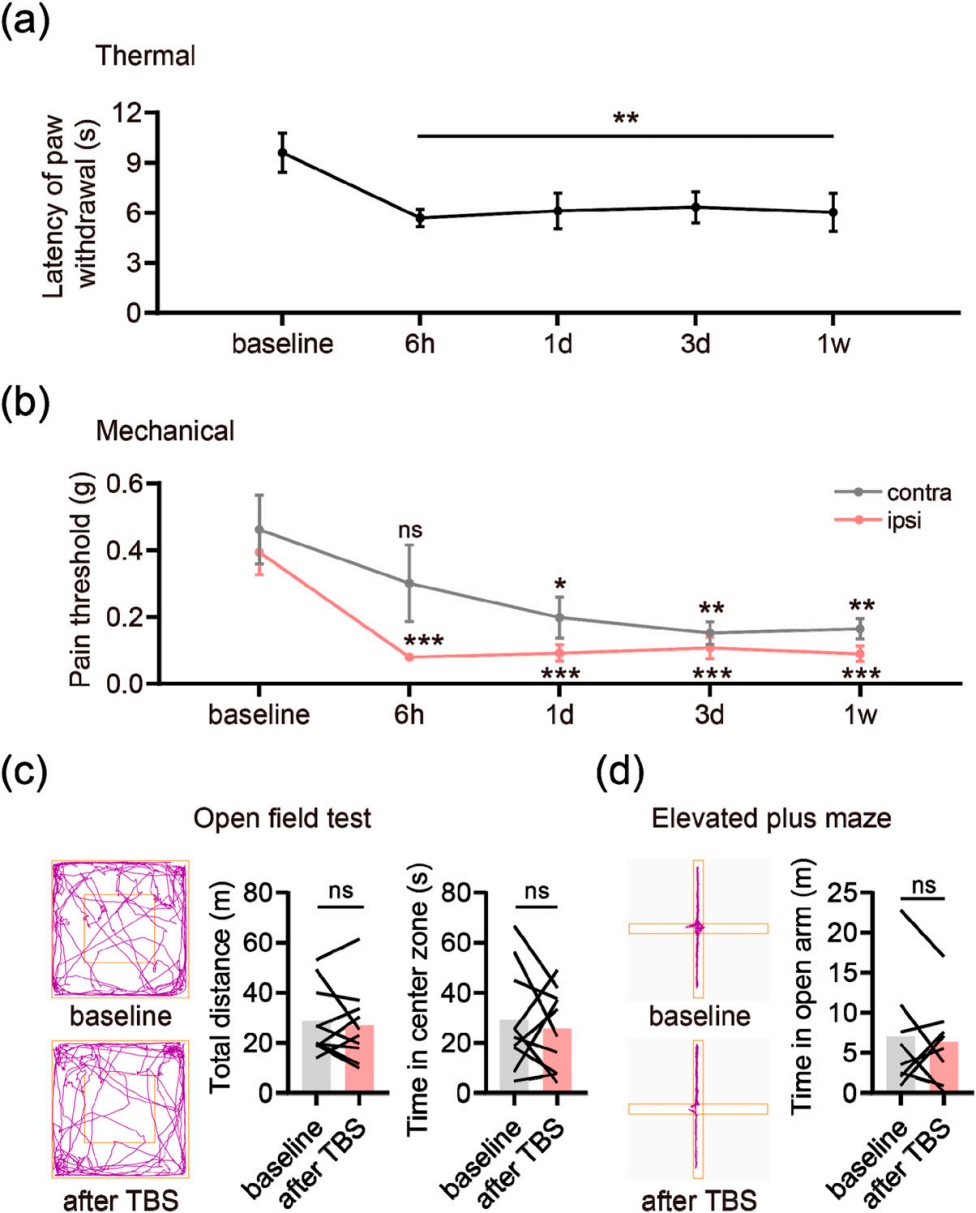
*mTH-ACC synaptic efficacy correlated with pain and anxiety related behaviors*. (a), The latency preceding paw withdrawal or licks on a 48°C plate following optical TBS measured up to 1 week post-TBS (n = 6 mice, **p < 0.01, paired *t*-test for each value comparison to baseline). (b), The pain threshold of the ipsilateral or contralateral hindpaw in response to different Von frey filaments pre- and post-TBS respectively measured up to 1 week post-TBS (n = 6 mice, *p < 0.05, **p < 0.01, ***p < 0.001, paired *t*-test for each value comparison to baseline). (c), The time in the center zone and total distance in the open field test following optical TBS (n = 10 mice, paired *t*-test for each value comparison between pre- and post-TBS). (d), The time in the open arm in the elevated plus maze test following optical TBS (n = 8 mice, paired *t*-test for each value comparison between pre- and post-TBS).

In addition to contributing to pain perception, recent studies have indicated that ACC activation also facilitate spinal nociceptive transmission through direct and indirect top-down modulation.^37,38^ To examine possible effects of potentiated mTH-ACC LTP on behavioral sensory responses, we measured behavioral responses to nociceptive thermal and mechanical nociceptive stimuli in freely moving mice. We found that behavioral responses in the hot plate test were significantly facilitated (n=8 mice; p<0.001 at 48°C compared to before TBS, unpaired *t*-test for average value comparison; Figure 4(a)). This effect was first observed at 30 min after the induction of LTP and lasting for up to 1 week (n = 6 mice, p < 0.001, paired *t*-test for average value comparison to baseline). Similar results were found with behavioral responses to mechanical Von-Frey stimuli (n=8 mice; ipsilateral hindpaw, p=0.002 and contralateral hindpaw, p=0.7 compared to before TBS, unpaired *t*-test for average value comparison; Figure 4(b)), and also lasting for up to 1 week (n = 6 mice, p < 0.001, paired *t*-test for average value comparison to baseline).

Previous reports have linked pre-LTP in the ACC to chronic pain-related anxiety-like behaviors.^9,16^ We thus decided to perform the open field test (OFT) and elevated plus maze (EPM), to assess anxiety-like behaviors in mice before and after the induction of TBS-LTP. In the OFT, we found that there was no significant difference in total movement distance between baseline and after TBS LTP (n=10 mice, p=0.872, paired t-test for each value comparison between pre- and post-TBS, Figure 4(c)), indicating that TBS-induced LTP in the mTH-ACC pathway did not alter the basal locomotor function of freely moving mice. Moreover, the percentage of time that mice spent in the central zone of the open field chamber, a core readout for anxiety-like behaviors, showed no statistically significant change after TBS-LTP (n=10 mice, p=0.615, paired t-test for each value comparison between pre- and post-TBS). Consistent with the OFT findings, results from the EPM test further confirmed that TBS-LTP in the mTH-ACC pathway did not trigger significant changes in anxiety-like behaviors. Neither the percentage of time spent in the open arms nor the number of entries into the open arms differed significantly between pre- and post-TBS conditions (n=8 mice, p=0.793 for open arm duration percentage, p=0.824 for open arm entry number, paired t-test for each value comparison between pre- and post-TBS, Figure 4(d)). Taken together, these results demonstrate that TBS-induced LTP in the mTH-ACC pathway is sufficient to drive robust nociceptive sensitization in both thermal and mechanical modalities, but does not induce significant anxiety-like behaviors or affect basal locomotor activity in adult mice under physiological conditions.

### The mTH-ACC pathway significantly altered after peripheral inflammation

**Figure 5.**
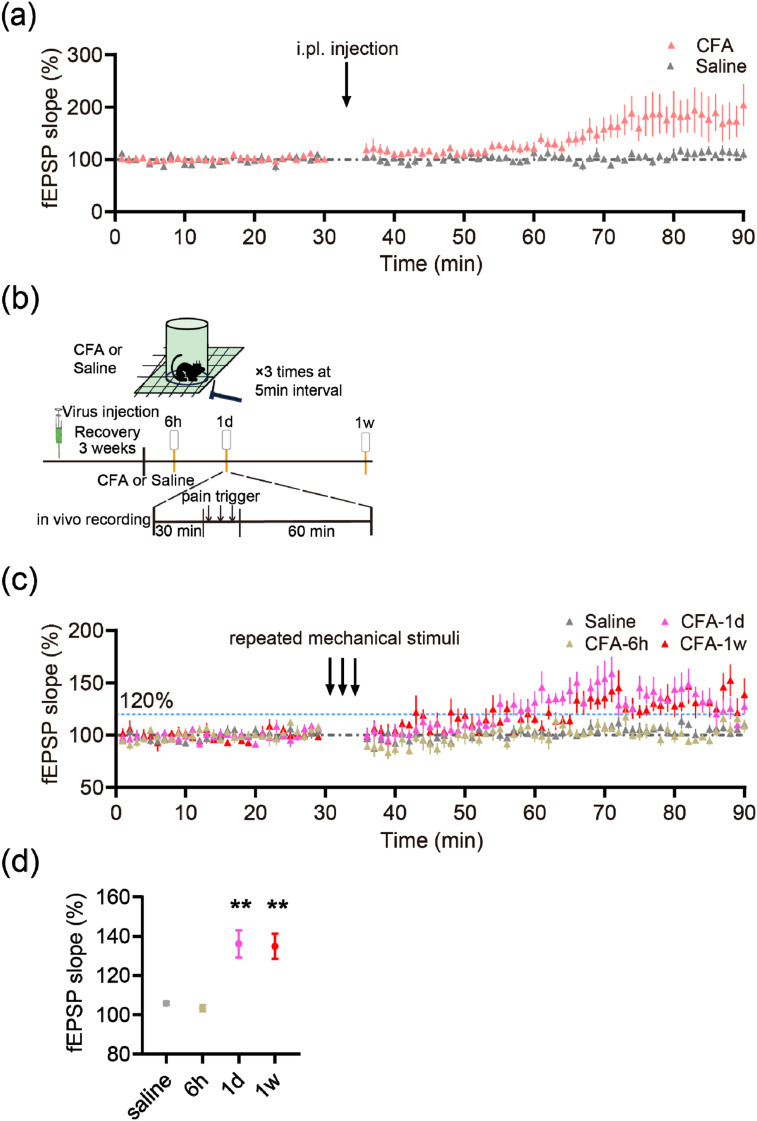
*mTH-ACC synaptic efficacy potentiated in inflammation pain*. (a), Plot of baseline normalized fEPSP optically evoked responses (n = 5 mice) after CFA intraplantar (i.pl.) injection into the plantar surface of the hind paw. Error bars show s. e.m.(b), Schematic of the repeated application of mechanical stimulation pain by Von frey filaments after CFA or saline treatment. (c), Plot of baseline normalized fEPSP optically evoked responses (n = 6 mice) after repeated pain stimuli at 6 hours, 1 day, 3 days after CFA or saline treatment. Error bars show s. e.m. (d), Normalized optoevoke-fEPSP in mTH-ACC pathway (normalized to baseline period) recorded after repeated pain stimuli (n = 5 mice, **p < 0.01, one sample *t-*test for average value comparison).

Previous studies in vitro and in vivo (anesthetized rats) show that peripheral injury triggered LTP in the ACC and potentiated synaptic responses in the ACC.^16,20,22,32^ To test if synaptic transmission of the mTH-ACC pathway is potentiated after the injury, we injected complete Freund’s adjuvant (CFA) into the plantar surface of the hind paw and recorded fEPSPs to stimulation of the mTH-ACC. We found that fEPSPs potentiation were observed at early time points after CFA treatment (Figure 5(a)). It is known that ACC responses can be sensitized for a long period of time, and its responses to weak sensory stimuli can be enhanced after injury.^22^ We further examined ACC fEPSPs to repeated mild mechanical stimuli after CFA. After the CFA injection, we applied the repeated mechanical stimulation pain at the plantar surface of the hind paw at different time points (each for 1 minute and trigger for 3 times at 5 min interval) by Von frey filaments (1.4g) (Figure 5(b)). We found that fEPSPs were significantly increased by the stimulation in CFA group as compared to saline-treated animals (n = 6 mice; Figure 5(c) and(d)).

### Depotentiation or LTD of the mTH-ACC pathway

**Figure 6.**
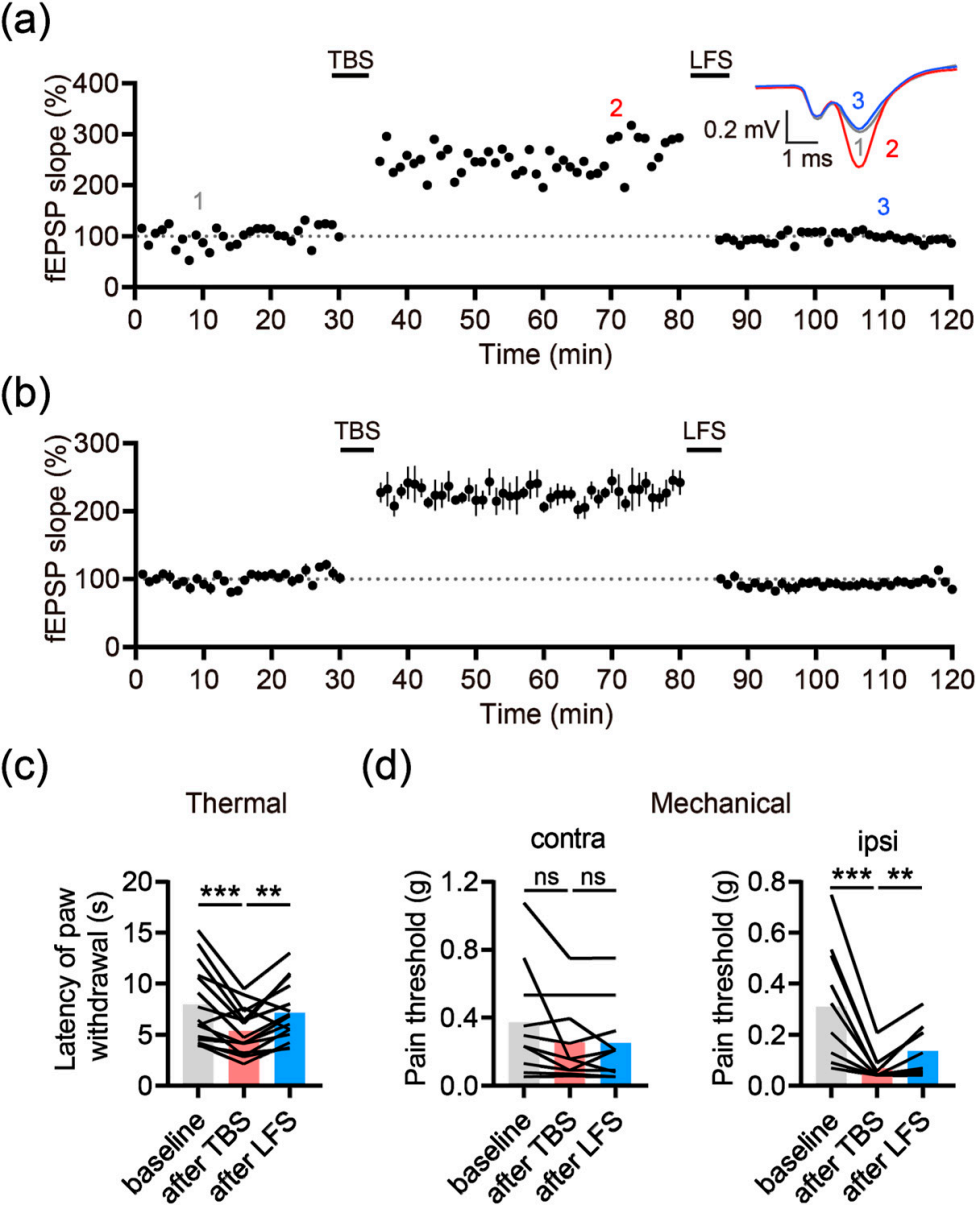
*Low frequency stimulation produced LTD in the mTH-ACC pathway*. (a), Top, representative fEPSP traces recorded at the different time points indicated by the numbers in the curve below. Bottom, the example curve of fEPSP slope induced by TBS and LFS blue light stimulation. (b), Plot of average of 6 experiments of field EPSP slope (n = 6 mice, normalized to baseline period) before and after indicated stimulation. Scale bars: 0.2 mV, 1 ms. Error bars show s. e.m. (c), The latency preceding paw withdrawal or licks on a 48°C plate following optical TBS and LFS after saline injection (n = 12 mice, **p < 0.01, ***p < 0.001, paired *t*-test for average value comparison). (d), The pain threshold of the ipsilateral or contralateral hindpaw in response to different Von frey filaments after TBS and LFS respectively (n = 10 mice, **p < 0.01, paired *t*-test for average value comparison).

In vitro brain slice experiments have revealed that ACC plasticity is biphasic, while TBS induces LTP, low-frequency stimulation induces LTD.^39,40^ It is unclear if LTD may depotentiate LTP in freely moving mice in vivo. We applied low frequency stimulation (LFS) to the mTH-ACC pathway at one hour after TBS induced LTP. We found that LFS effectively reversed TBS-LTP in adult mice (n=6 mice, P<0.001 as compared with 217%±13.5% of the baseline after TBS, P<0.001 as compared with TBS average value after TBS-LFS, P=0.875 as compared with 94.5%±2.9% of the baseline, mean±SEM; Figure 6(a) and (b)). Behaviorally, we found that LFS in the mTH-ACC pathway significantly reversed both thermal pain in hot plate (n=12 mice; p=0.003 at 48°Ccompared to after TBS, paired *t*-test for comparison; Figure 6(c)) and mechanical pain thresholds (n=12 mice; ipsilateral hindpaw, p=0.007 and contralateral hindpaw, p=0.883 compared to after TBS, paired *t*-test for comparison; Figure 6(d)).

## Discussion

By using in vivo electrophysiological recording and optogenetic stimulation, we demonstrate that the thalamus-ACC projection is highly plastic and undergo LTP in adult freely moving mice. LTP is long-lasting, and persists for at least days to weeks after the induction. Behaviorally, this potentiation can induce facilitation of behavioral responses to noxious stimuli without any peripheral injuries, suggesting that central plasticity alone is sufficient to cause behavioral sensitization in the freely moving conditions. These findings support the hypothesis that ACC plasticity including the projection from the thalamus play important roles in behavioral sensitization caused by peripheral tissue injuries. Our studies provide strong direct evidence for the thalamus-ACC projection in behavioral sensitization. To further support this, depotentiation or LTD after LTP is able to reverse behavioral sensitization, indicating that synaptic strength between the thalamus and ACC can regulate the nociceptive threshold of behavioral responses to noxious stimuli. Although we did not detect changes in anxiety-like behaviors, it is likely due to possible threshold-related (that anxiety may be triggered at greater intensities) or the anxiety is mediated through other projection pathways and network. Future studies are clearly needed to explore this question. In addition to pain perception, recent studies suggest that ACC may also affect behavioral responses to noxious stimuli by top-down modulatory systems.^37,38^ Although we did not investigate this possibility in the present study, the current animal models will provide a powerful model for such investigation in future.

### The induction of the mTH-ACC LTP both in vitro and in vivo

Recent evidence has suggested that neurons in the ACC are likely to receive multiple projections from other cortical and subcortical areas (see Introduction), and many of these projections are likely to be excitatory and glutamate serves as a key transmitter. Thus, it is difficult to determine the inputs of synaptic responses in brain slices experiments in vitro when focal electrode is used.^9,16,31^ The use of virus and optogenetic approaches have helped us to selectively activate projection fibers in both in vitro and in vivo. For example, Li et al. showed that activation of callosal projection fibers induced fast excitatory responses in the ACC neurons in vitro.^28^ In our recent study, Xue et al. reported that activation of thalamic afferents fibers optogenetically caused monosynaptic excitatory responses in ACC pyramidal cells, and these responses were blocked by AMPA receptor antagonist.^18^ In this study, we found that TBS in the thalamus projection fibers triggered LTP in the ACC slices of adult mice. This LTP share similar onset and duration as previously reported by electrical stimulation. Furthermore, we found that TBS in the mTH-ACC pathway is able to recruit silent responses, consistently with previous reports of silent responses are recruited by TBS induced LTP and chemically (forskolin).^33,34^

One major limitation of brain slice experiment for LTP is the duration of LTP recorded. It is usually to be 1 hr in case of whole-cell patch experiments, and several hours in the field recording.^31,32^ The use of MED recording system has significantly improved the recording time, and it lasted for more than 6 hours.^40,41^ In the present study, using the recording from freely moving mice, we are able to record LTP induced by TBS at the mTH-ACC pathway for several days to weeks. These findings provide the direct evidence for the existence of long-lasting LTP in the thalamus-ACC synapses of adult animals, and such LTP could play cortical roles in chronic pain and its related emotional disorders.^9,12^

### TBS induced mTH-ACC LTP contribute to pain hypersensitivity but not anxiety

Previous studies using brain slice electrophysiology, pharmacology, and behavioral measurement have consistently demonstrate that excitatory synapses play important roles in chronic pain and its related anxiety and depression.^12,16^ Previous in vitro experiments demonstrate that TBS induced LTP in the ACC is primarily post-synaptic. While NMDA receptor contributes to the induction, postsynaptic regulation of AMPA receptors contributes to the expression of LTP.^32,33^ In this study, we found that TBS-LTP leads to behavioral sensitization, while depotentiation of LTP produce reversed effect. Interestingly, anxiety-like responses are not affected. These findings suggest that TBS induced LTP may mainly contribute to sensitization of behavioral responses to sensory stimuli, while emotion related anxiety responses are mediated by other pathways. We cannot completely rule out the possibility that TBS-LTP’s contribution to anxiety maybe pattern or intensity-dependent, future studies are needed to investigate this possibility. It is quite possible that the interaction between the ACC and amygdala may play more roles in injury-related anxiety.^42^

### mTH-ACC LTD

One key function for LTD in central synapses is to reset synaptic tone after LTP or to depotentiate LTP.^43,44^ In case of memory, it has been proposed that LTD can be a possible mechanism for forgetting.^45^ In the present study, we are able to induce LTD of potentiated mTH-ACC synapses in freely moving animals, providing the direct evidence that LTD can depotentiate LTP induced by TBS. Functionally, such LTD is able to reduce behavioral sensitization produced by LTP at the same pathway, indicating that the mTH-ACC pathway is highly plastic in adult animals, and LTD can act as a key mechanism to re-set potentiated synaptic responses in vivo. Consistently, recent studies using chemicals injected into the ACC to restore LTD in vitro has been found to produce analgesic effects.^46^

In summary, our studies provide the direct evidence for adult LTP at the key central relay pathway, the thalamus to the ACC both in vitro and in vivo. Behavioral studies show the causal correlation of this potentiation with behavioral responses to noxious stimuli. Together, these findings fill the gap of our understanding of plasticity in chronic pain, and will facilitate future treatment of patients with chronic pain.

## Materials and methods

### Subject

Adult (8-16 weeks) male C57BL/6J mice were used for experiments. Mice were maintained under a 12-hour light/dark cycle at 22-25 °C and given ad libitum access to tap water and standard chow. All animal use procedures were conducted in accordance with the guidelines of the Animal Advisory Committee at Fujian Medical University, following the US National Institutes of Health Guidelines for the Care and Use of Laboratory Animals.

### Stereotaxic surgery

Mice at 8-12 weeks of age were anesthetized with isoflurane (Sigma, USA) and secured in a stereotaxic frame (RWD). We used a AAV2/9-hChR2(H134R)-mCherry driven by the excitatory neuron-specific CaMKIIα promoter (Vigenebio, China, 1.5 ×10^13^particles/ml) or AAV2/9-mCherry virus (Vigenebio, China, 2.3 ×10^13^particles/ml) for control. AAV viruses were injected at a speed of 30-40 nl/min into mTH. The injection coordinates for the mTH were anterior-posterior (AP): -1.5 mm; medial-lateral (ML): 0.3 mm; dorsal-ventral (DV): 3.3 mm. A volume of 250 nl of AAV viruses was injected into the right side of mTH. At the end of the injection, the pipet remained at the site for 5 min to allow for diffusion of the virus. After neurosurgery, the mice generally recover for 3-5 days according to their recovery status before further experiment.

### Optrode implantation

The recording optrode consisted of an electrode made of nickel-cadmium wires (0.0014 inch, California Fine Wire) and an optical fiber. The tip of the optical fiber was 0.3-0.5 mm above the tip of the electrodes. The optrode wire tips were gold-plated (using a gold-plating solution, NeuraLynx) to adjust the impedance to 500-800 kΩ. The optrodes were implanted at least 2 weeks after AAV viruses injection, and lowered into the brain targeting the right side of ACC (AP: 1.0, ML: 0.4, DV: 1.3 mm from dura). Neuronal signals were referenced to two connected skull screws (placed above the prefrontal cortex and cerebellum). Then layers of dental cement were applied to affix optrodes to the skull. After the cement dried and full recovery, we transferred the animal to their homecage. The mice generally recover for 3-5 days before signal recording and optical stimulation.

### Multi-channel field potential recording

Multi-channel fEPSPs were recorded using the MED64 multichannel recording system (Panasonic Alpha-Med Sciences). The MED64 probe (P515A; 64 square planar microelectrode arrays arranged in 8 × 8 mode; interpolar distance, 150 μm; Panasonic Alpha-Med Sciences) requires a hydrophilic coating prior to the experiment, with a method similar to that in our previous studies. Briefly, the probe was treated with 0.1% polyethyleneimine (Sigma-Aldrich) dissolved in 25 mmol/L borate buffer (pH 8.4) and left at room temperature overnight. The probe surface was then carefully rinsed three times with sterile distilled water to remove any potentially harmful substances and avoid affecting the brain slices. Hydrophilic treatment facilitated better attachment of brain slices to the MED64 probe. The incubated brain slices were sticked onto the MED64 probe as closely as possible (Figure 1(a)). After positioning a slice, it was carefully secured with a mesh and an anchor (Warner Instruments, Harvard Apparatus) to ensure the stability during the experimental recording. Throughout the experimental period, slices were continuously perfused with fresh artificial cerebrospinal fluid (ACSF) oxygenated (95% O_2_ and 5% CO_2_) at a flow rate of 2–3 ml/min. Slices were restored for at least 1 h before recording. A stimulus site was selected from 64 planar microelectrodes, and a constant current pulse of 0.2 ms duration was delivered to the superficial layer (Layers 2-3) of the ACC slices. In optogenetic experiments, the current pulse was replaced by a blue light pluse of 0.2-0.5 ms duration (10-30mW). Stimulus intensity was adjusted to evoke a half-maximal fEPSP in nearby channels and to recruit as many channels as possible. The fEPSPs recordings were sampled every 1 min, with averages calculated every 4 min (Figure 1(b), (c), (e)) or every 2 min (Figure 2(b) and (d)) in offline analysis. A stable baseline response was recorded for at least 20 min (with a single channel response fluctuation between 5-10%), after which LTP was induced by using current or light based TBS (five bursts, each consisting of four pulses at 100 Hz, delivered at 200 ms intervals; repeated five times with an interval of 10 s, 4 × 5×5). After LTP induction, fEPSP responses were continuously recorded for at least 2 h.

### In vivo electrophysiology recording

For optical stimulation, the optical fiber was connected to a 473 nm laser (Inper, China). 1 ms light pulses at 0.033 Hz was delivered to evoke field EPSP. Neural signals were recorded using a Digital Zeus system with HS-18-MM headstage pre-amplifier (Bio-Signal Technologies, China). Recorded signals were filtered between 0.6 and 10 kHz and sampled at 30 kHz. For optical LTP experiment, only after baseline was stable for at least 30 min, optical TBS (theta burst stimulation, five bursts of 4X 1 ms 100Hz pulses given at 200 intervals within 1second, five trains of 1 s 5X bursts with 10 s inter-train-interval) or the optical LFS (low frequency stimulation, 1Hz for 20 minutes) was delivered at the recording site, followed by fEPSP recording for at least another 1 hour. Each fEPSP was normalized to field potentials 10 ms before optical stimulation. Slope of the normalized fEPSP was calculated as described (see Figure 1). All data were analyzed with custom written MATLAB program.

### Von Frey withdrawal threshold test

Nine von Frey filaments (Aesthesio), ranging from 0.04 to 4.0 g were used to assess mechanical withdrawal thresholds. Filaments were applied perpendicular to the central hind paw surface with sufficient force to cause a slight bending of the filament, using 0.6 g von Frey fiber as a start. A positive response was characterized by a rapid withdrawal of the paw away from the stimulus fiber within 6-8s. The up-down method was used to determine the mechanical threshold (50% withdrawal threshold).^47^

### Hot plate test

Hot plate test was used to determine the thermal threshold. Mouse was placed on a hot plate at 48 °C (10cm×10cm) surrounded by an animal container (25 cm×10 cm×10 cm). Elevation of the hind paw, licking or jumping were defined as responses to the thermal stimulus. The latency to a nociceptive response was defined as the thermal pain threshold. The cutoff time was 60s to avoid burns injury in 48 °C hot plate. For the pain-related behavioral assessments following optical theta-burst stimulation (TBS) or low-frequency stimulation (LFS).

First, baseline pain thresholds were established through sequential testing. Mice were placed in transparent containers on a wire mesh platform and allowed 30 minutes for environmental acclimatization, during which their plantar surfaces were intermittently stimulated with Von Frey filaments. Quantitative sensory testing was then performed using Von Frey filaments applied to both hind paws, followed by a two-stage hot plate test (48°C) with 10-minute inter-test intervals.

Following baseline measurements, mice received TBS protocol administration (50-second laser stimulation via implanted optical fiber). Subjects were then returned to the testing apparatus for 30-minute re-acclimatization before repeating the full behavioral assessment sequence.

Subsequently, LFS protocol was administered (15-minute laser stimulation) followed by identical 30-minute acclimatization and behavioral retesting procedures. All experimental manipulations were conducted under standardized laboratory conditions with ambient temperature maintained at 22±1°C.

### Open field test

In the open field test, mice were gently placed in the center of an open-field chamber (50 ×50×40 cm) and allowed to freely explore the environment for 10 min. Animal locomotion and movement trajectories were tracked and analyzed using ANY-maze software. The percentage of time spent in the central zone was recorded to assess anxiety-like behavior.

### Elevated plus maze

The elevated plus maze consisted of two open arms and two enclosed arms, forming a cross-shaped maze elevated 50 cm above the floor. All four arms were identical in size (35 cm × 5 cm). Each mouse was placed in the central square platform (5 cm × 5 cm), facing an open arm, and allowed to freely explore the maze for 5 min. The time spent in the open arms and the number of entries into the open arms were recorded to assess anxiety-like behavior.

## Histology

Tissue collection and processing. Animals were deeply anaesthetized with intraperitoneal injection of tribromoethanol, then transcardially perfused with phosphate-buffered saline (PBS) followed by 4% formaldehyde in PBS. Brains were carefully dissected and post-fixed in 4% formaldehyde at 4°C overnight, and transferred to 15 and 30% sucrose solution at 4°C for dehydration. Tissues were then frozen in Optimum Cutting Temperature compound (OCT; product code: 4583, Tissue Tek) and sectioned using a cryostat (Leica). Brains were sectioned at 40 μm and stored in PBS at 4°C if used immediately. For longer storage, tissue sections were placed in glycerol-based cryoprotectant solution and stored at -20°C.

## Supplemental Material

Supplemental material - Biphasic synaptic plasticity of the medial thalamic-cingulate pathway contributes to pain hypersensitivity in vivo Supplemental material for Biphasic synaptic plasticity of the medial thalamic-cingulate pathway contributes to pain hypersensitivity in vivo by Shen Lin, Shun Hao, Yi Wu, Weiqi Liu, Jihua Wang, Le Li, Wanfen Liu, Wucheng Tao, Min Zhuo in Molecular Pain.
