# Supplementary material for: Biphasic synaptic plasticity of the medial thalamic-cingulate pathway contributes to pain hypersensitivity in vivo
Source: Mol Pain. 2026 Jul 15;22:17448069261466896. doi: 10.1177/17448069261466896 (PMC13447995; doi:10.1177/17448069261466896)
Supplement: Supplementary file 1 [file sj-pdf-1-mpx-10.1177_17448069261466896.pdf]

## Supplement figures

**Figure. S1 (Related to Figure 3)** *Viral targeting in mTH and optical fiber/optrode placement in ACC*

(a), Brain slices from a mouse with AAV-CaMKII-hChR2(H134R)-mCherry virus injected into mTH. MDL: lateral part of mediodorsal thalamus; MDC, central part of mediodorsal thalamus; MDM, medial part of mediodorsal thalamus. Red, mCherry fused to ChR2. Dashed white lines are boundaries of subregions. (b), Overlay of ChR2-mCherry expression in mice which received light stimulation for electrophysiological recording or behavioral tests (n=18 mice). Red, viral targeting. (c), Placement of all optrodes in ACC for eletrophysiological recordings and optical fibers for behavioral tests.

**Figure S1**

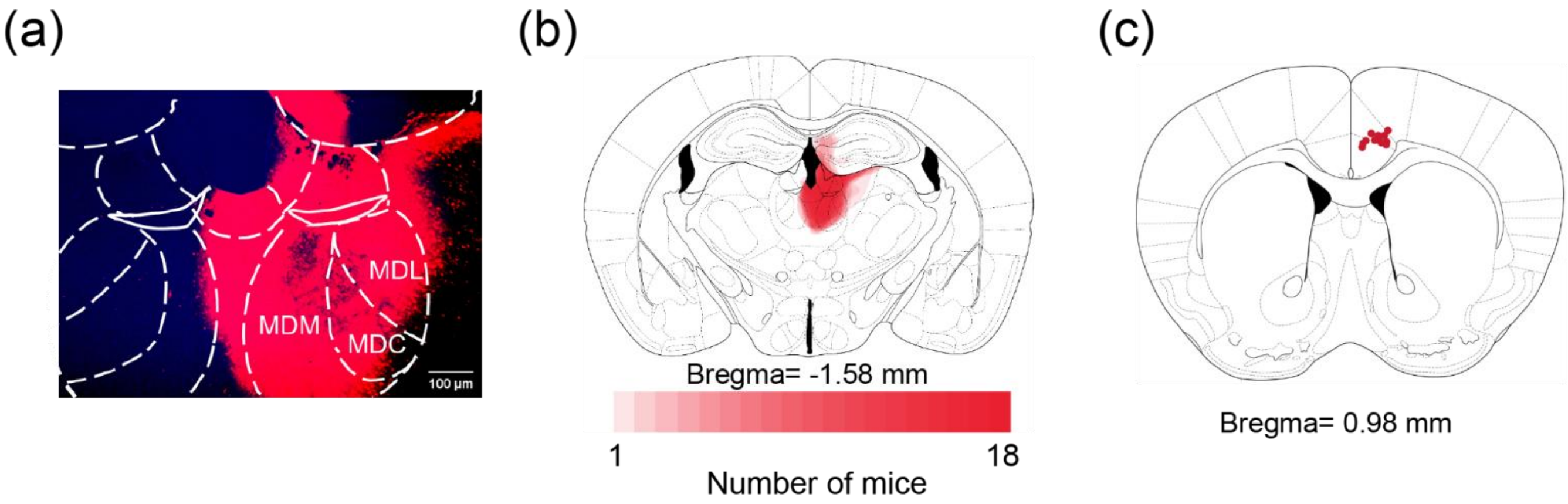
(a)
MDL
MDM
MDC
100 µm
(b)
Bregma= -1.58 mm
1
18
Number of mice
(c)
Bregma= 0.98 mm
